# Landscape of Exhausted T Cells in Tuberculosis Revealed by Single-Cell Sequencing

**DOI:** 10.1128/spectrum.02839-22

**Published:** 2023-03-14

**Authors:** Jiahui Pan, Xinyue Zhang, Jianting Xu, Zecheng Chang, Zhuoyuan Xin, Guoqing Wang

**Affiliations:** a Key Laboratory of Zoonosis Research, Ministry of Education, College of Basic Medical Science, Jilin University, Changchun, China; b The First Hospital of Jilin University, Changchun, China; Shenzhen University School of Medicine

**Keywords:** single-cell sequencing, tuberculosis, exhausted T cells, transcriptome signature

## Abstract

Tuberculosis, a contagious bacterial infection caused by Mycobacterium tuberculosis, is a substantial global health problem, impacting millions of lives annually. Exhausted T-cell signatures are critical for predicting clinical responses to tuberculosis infection. To obtain a panoramic transcriptional profile of T cells, we performed single-cell RNA-sequencing analysis of CD4^+^ T and CD8^+^ T cells isolated from peripheral blood mononuclear cells of healthy individuals and patients with tuberculosis. We identified seven subsets in CD8^+^ T cells and eight subsets in CD4^+^ T cells and elucidated the transcriptomic landscape changes and characteristics of each subset. We further investigated the cell-to-cell relationship of each subgroup of the two cell types. Different signature genes and pathways of exhausted CD4^+^ and CD8^+^ T cells were examined. We identified 12 genes with potential associations of T-cell exhaustion after tuberculosis infection. We also identified five genes as potential exhaustion marker genes. The CD8-EX3 subcluster in CD8^+^ T-exhausted cells was identified as an exhaustion-specific subcluster. The identified gene module further clarified the key factors influencing CD8^+^ T cell exhaustion. These data provide new insights into T-cell signatures in tuberculosis-exhausted populations.

**IMPORTANCE** Identifying the changes in immune cells in response to infection can provide a better understanding of the effects of Mycobacterium tuberculosis on the host immune system. We performed single-cell RNA-sequencing analysis of CD4^+^ T and CD8^+^ T cells isolated from peripheral blood mononuclear cells of healthy individuals and patients with tuberculosis to reveal the cellular characteristics. Different signature genes and pathways of exhausted CD4^+^ and CD8^+^ T cells were examined. These will facilitate a more comprehensive understanding of the onset and underlying mechanism of T-cell exhaustion during active *Mtb* infection.

## INTRODUCTION

Tuberculosis (TB) has erupted as a severe global health problem, claiming millions of lives annually (1). Adaptive immune responses mediated by T cells are important for the controlling of Mycobacterium tuberculosis (*Mtb*) infection (2). In continuous *Mtb* infection, prolonged overexposure to antigens can cause T cells to gradually lose their effector functions and exhibit an exhausted phenotype, called T-cell exhaustion, which cannot easily be recovered (3). T-cell exhaustion manifests as inactivated T-cell proliferation, secretion of inhibitory cytokines, and reduced interferon (IFN)-c production (4 to 6). This cell exhaustion state may affect the clinical manifestations of TB infection, such as disease severity and prognosis. Exhausted T cells also have specific effectiveness and reversibility characteristics. Therefore, a detailed investigation of T-cell exhaustion in TB infection can provide new targets and treatment strategies.

Although transcriptome sequencing can reveal differences in gene expression profiles (7), it cannot explain the characteristics of specific subpopulations in cells. With the progress in RNA sequencing, single-cell RNA sequencing (scRNA-seq) can be used to analyze the transcriptomic profiles of individual cells, revealing cellular identity and spatial organization in complex heterogeneous immune populations (8 to 10). Therefore, it is feasible to use scRNA-seq to determine the dynamic changes in T-cell subsets during TB infection. Previous studies revealed that anti-T-cell exhaustion therapy may reduce the recurrence rate of the disease, thereby offering a new treatment strategy (11, 12). Certain proportions of T cell subsets are considered to control the host immune response to disease (13). Some T-cell subsets have been suggested as markers for different disease states (14). However, the mechanisms of functional regulation and the efficacy of immune cell responses are heterogeneous and complex. The nature and function of cellular exhaustion patterns in patients with TB remain unclear.

In this study, we used scRNA-seq to comprehensively characterize the transcriptome profile of cellular subpopulations of CD4^+^ and CD8^+^ T cells isolated from the peripheral blood of patients during TB infection. We also calculated the connectivity among the cell subsets. In particular, we elaborated on the composition and gene expression pattern heterogeneity of exhausted cell subsets. Our study revealed the exhaustion characteristics and key factors of the two exhausted cell subsets, which can facilitate a more comprehensive understanding of the onset and underlying mechanism of T-cell exhaustion during active *Mtb* infection.

## RESULTS

### T-cell transcriptional profiling among individuals with Mtb infection.

**FIG 1:**
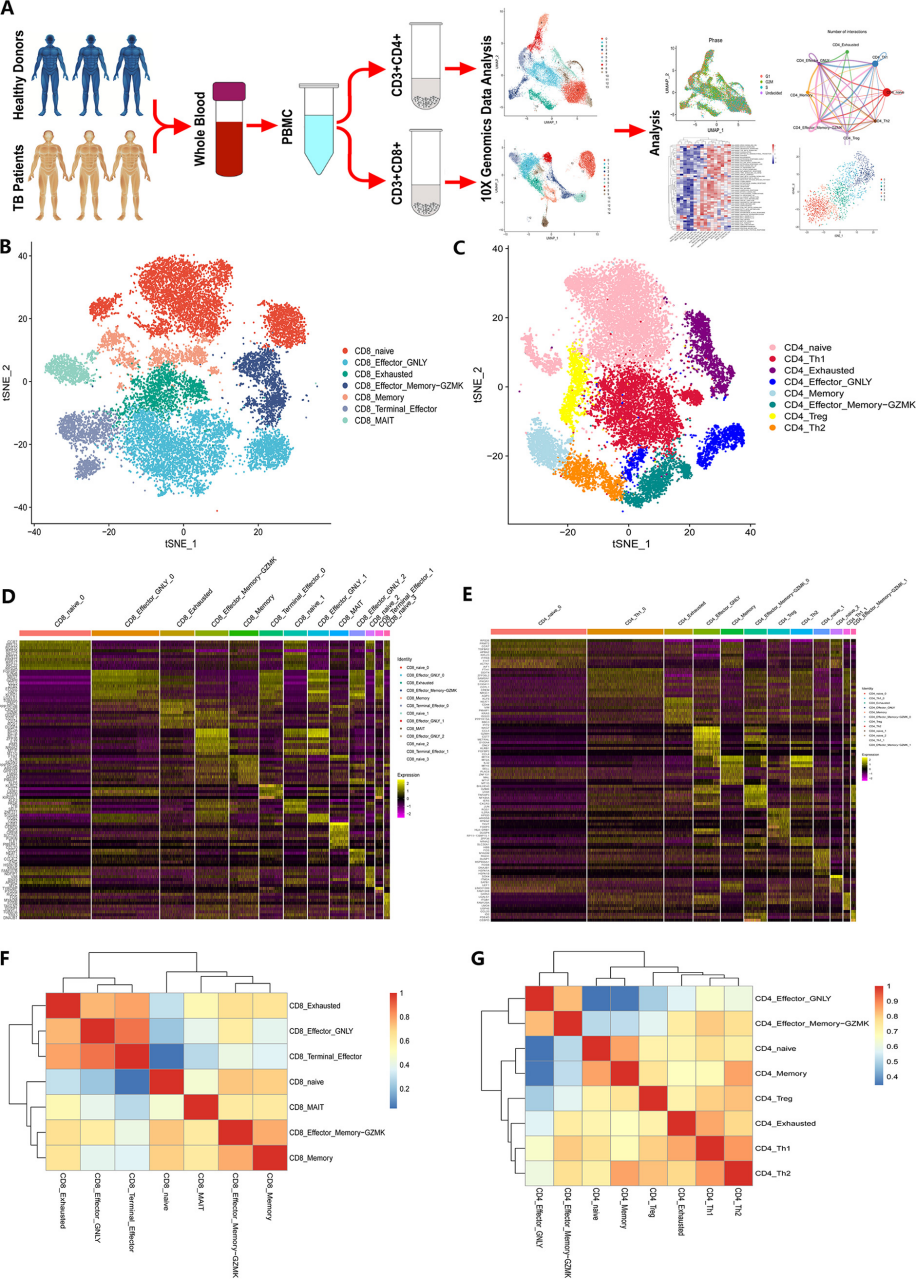
Single-cell transcriptomic clustering of CD4^+^ and CD8^+^ T cells from patients with active TB and healthy donor individuals. (A) Schematic workflow design for scRNA-seq. CD4^+^ and CD8^+^ T cells were purified from PBMCs collected from three patients with active TB and three healthy donors. A 10× genomic scRNA-seq platform was used to perform transcriptomic profiling. (B and C) CD8^+^ (B) and CD4^+^ (C) T-cell subset population features. The t-SNE projection was used to visualize the nine CD8^+^ T cell subsets. Each dot represents a single cell colored according to the cell clustering information. (D and E) Heatmaps showing the top marker genes across each CD8^+^ (D) and CD4^+^ (E) T-cell cluster. (F and G) Heatmaps showing the pairwise Pearson correlation coefficients of T-cell subsets for CD8^+^ T cells (F) and CD4^+^ T cells (G).

First, 10× genomic scRNA-seq was performed on 50,236 CD4^+^ and CD8^+^ T cells purified from peripheral blood mononuclear cell (PBMC) suspensions of three patients with TB and three healthy donors (Fig. 1A, Tables S1 and S2 in the supplemental material). After quality control, 39,444 cells were selected for subsequent analyses (Fig. S1 and S2). Among these, 13 CD8^+^ T-cell clusters (Fig. S3A, C, E) and 12 CD4^+^ T-cell clusters (Fig. S3B, D, F) were identified through unsupervised clustering analysis using Seurat 4.0. According to the distinct expression patterns of canonical T-cell biomarkers, we annotated these clusters into seven CD8^+^ T-cell subsets (Fig. 1B, D) and eight CD4^+^ T-cell subsets (Fig. 1C, E).

The main subsets were CD8^+^ naive cells, which expressed high *CCR7* and *LEF1* levels and CD8^+^ effector-GNLY cells, which expressed high levels of *GNLY*. We identified CD8^+^ terminal effector cells with a high expression of cytotoxic genes and a small amount of exhaustion genes, and CD8^+^ effector memory-GZMK cells displaying relatively high expression levels of the *GZMK* gene. We also identified CD8^+^ memory and CD8^+^ mucosa-associated invariant T cells (MAIT cells). Notably, we also identified exhaustion subsets among CD8^+^ T cells, which were characterized by a high expression of exhaustion markers, including *HLA-DRA*, *PDCD1*, and *TNFRSF9*.

For CD4^+^ T cells, the major T-cell subset was composed of naive cells with a high expression of *CCR7* and *FHIT*. We also detected regulatory T cells (Treg cells), which were characterized by a high expression of *FOXP3* and *RGS1*. Notably, some exhaustion markers were also highly expressed in Treg cell subsets. We also observed memory T-cell subsets and two T-helper (Th) cell types. CD4^+^ effector-GNLY cells were identified based on high expression of *GZMA*, *NKG7*, *GZMB*, *GZMH*, and *GNLY*. CD4^+^ effector memory-GZMK cells were also identified by a high expression of *GZMK* and a low expression of other cytotoxic genes. We further identified an exhausted T-cell subset with high expression levels of exhaustion markers (*HLA-DRA*, *PDCD1*, and *TNFRSF9*). Pearson correlation analysis was used to evaluate the relationships between subsets of CD8^+^ T cells (Fig. 1F) and CD4^+^ T cells (Fig. 1G). Naïve cells were less associated with effector cells for both CD4^+^ and CD8^+^ T cells. Collectively, these results clearly defined the composition of CD4^+^ and CD8^+^ T-cell subsets in patients with TB.

### Single-cell profiling revealed CD8^+^ and CD4^+^ T-cell subset heterogeneity.

**FIG 2:**
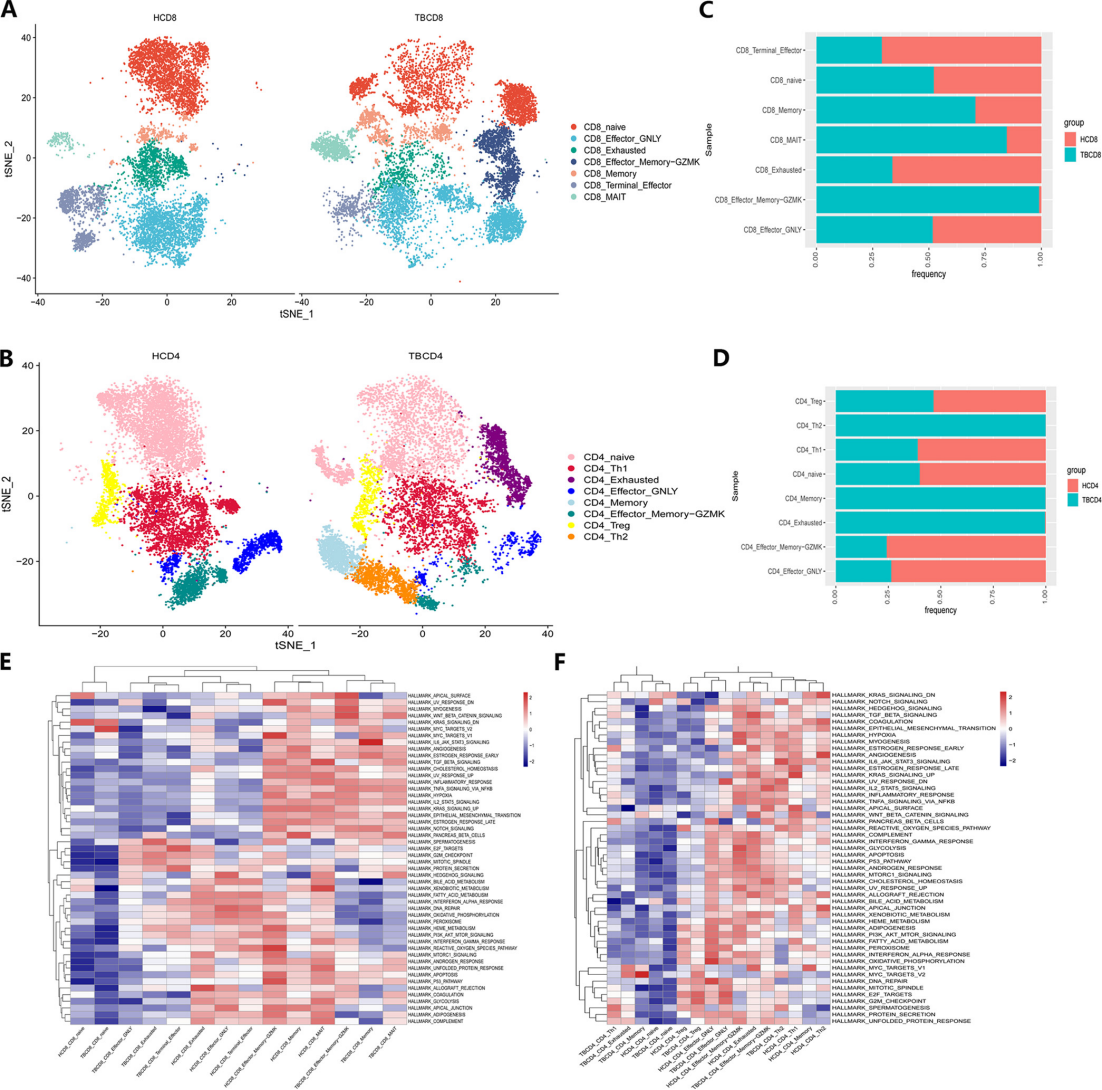
Heterogenic CD4^+^ and CD8^+^ T-cell subset characteristics in patients with active TB. (A and B) T-SNE diagrams of single-cell profiling for each CD8^+^ (A) and CD4^+^ (B) T-cell subset across the TB (left) and healthy donor (right) groups. Each dot represents a single cell colored according to the cell-clustering information. (C and D) Stacked bar plots for CD8^+^ (C) and CD4^+^ (D) T-cell proportions across the TB (blue) and healthy donor (red) groups. (E and F) Hallmark gene set-based GSVA heatmaps in each CD8^+^ (E) and CD4^+^ (F) T-cell subset across tuberculosis and healthy donor groups.

We evaluated the distribution of each CD4^+^ and CD8^+^ T-cell subset across patients with TB and healthy donors (Fig. 2A and B). The proportions of CD8^+^ effector memory-GZMK, memory, and MAIT cells were increased in patients with TB (Fig. 2C), along with increased proportions of CD4^+^ Th2, memory, and exhausted T cells (Fig. 2D). In addition, cell cycle distribution analysis showed that the number of cells in the G1 phase in the majority of CD8^+^ T-cell subsets in patients with TB was relatively decreased (Fig. S4A to C). In particular, after *Mtb* infection, the cell cycle of CD4^+^ memory and Th2 cell subsets changed substantially (Fig. S4D to F).

We further explored the functions of these subsets using gene set variation analysis (GSVA) (Fig. 2E and F). Among the two kinds of cells, the activity of each pathway of naive cells was quite low. After *Mtb* infection, CD8^+^ effector memory-GZMK and CD8^+^ memory cells exhibited similarly low rates of fatty acid metabolism. CD8^+^ MAIT cells and CD8^+^-exhausted T cells exhibited poor immune and metabolic profiles, such as a downregulated IFN-α response. CD8^+^ terminal effector cells did not change significantly. In CD4^+^ T cells, the activity of each pathway of naive and memory cells was also relatively low. After *Mtb* infection, CD4^+^ Th1 cells exhibited low expression of oxidative phosphorylation and fatty acid metabolism. Treg cell subsets had weaker transcriptional and metabolic activities. Notably, CD4^+^-exhausted cells showed significantly downregulated Notch signaling and WNT-β catenin signaling pathways. Hedgehog signaling was also significantly downregulated, indicating a weaker inflammatory response and immune activity. The activity of CD4^+^ effector-GNLY cells and CD4^+^ effector memory-GZMK cells changed slightly. Taken together, these analyses demonstrated the high degree of cell subset heterogeneity in CD4^+^ and CD8^+^ T cells after *Mtb* infection.

### Communication patterns of each subset of CD8^+^ and CD4^+^ T cells.

**FIG 3:**
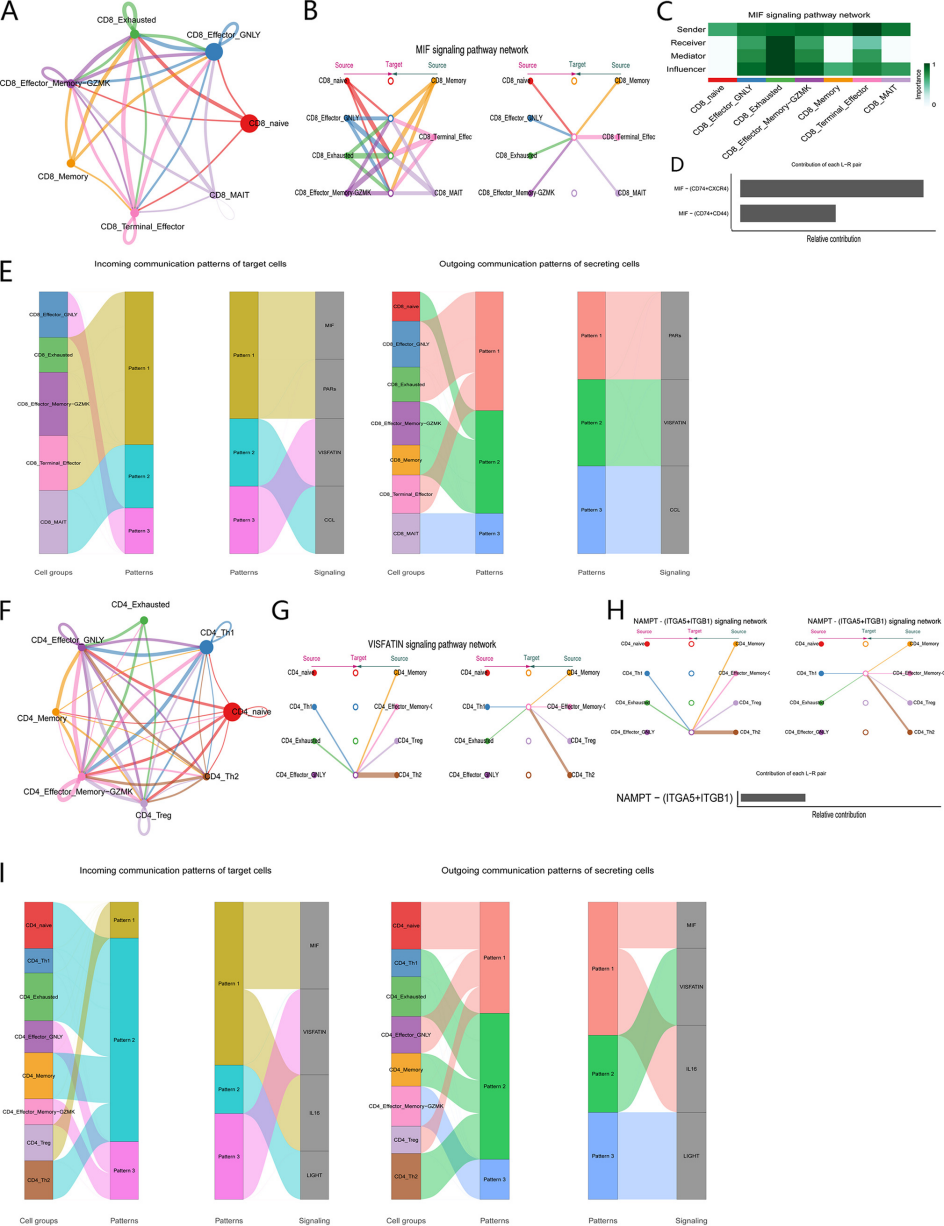
CellChat analysis of the communications between each cell subset of CD8^+^ and CD4^+^ T cells. (A) Cell communication among subsets of CD8^+^ T cells. (B) Hierarchical plot showing the inferred intercellular communication network for the MIF signaling pathway. (C) Heatmap showing the relative importance of each cell subset based on four network centrality measures of the MIF signaling network. (D) Relative contribution of each ligand–receptor pair to the MIF signaling pathway. (E) Inferred incoming (right) and outgoing (left) communication patterns of target and secreting cells of CD8^+^ T cells, respectively. (F) Cell communication among subsets of CD4^+^ T cells. (G) Hierarchical plot showing the inferred intercellular communication network for the VISFATIN signaling pathway. (H) Relative contribution of each ligand–receptor pair to the VISFATIN signaling pathway. (I) Inferred incoming (right) and outgoing (left) communication patterns of target cells and secreting cells of CD4^+^ T cells, respectively.

We conducted CellChat analysis for the CD8^+^ and CD4^+^ T cells to evaluate the communication patterns between each cell subgroup (Fig. 3A, F). In CD8^+^ T cells, the macrophage migration inhibitory factor (MIF) signaling pathway was the most common pathway among the subgroups, which revealed the complexity of the network (Fig. 3B). For exhausted cell subsets, both autocrine signaling and paracrine signaling patterns were evident (Fig. 3C). In all known ligand-receptor pairs, the MIF pathway was mainly controlled by the MIF ligand and CD74, CXCR4, and CD44 receptors (Fig. 3D). In CD4^+^ T cells, we observed the VISFATIN signaling pathway (Fig. 3G), and its network analysis was not redundant. This pathway can activate the NF-κB signaling transduction pathway and participates in immune regulation (15). Among all known ligand-receptor pairs, this is mainly controlled by the NAMPT ligand and the ITGA5 and ITGB1 receptors (Fig. 3H).

We further investigated how these multiple cell groups and signaling pathways coordinate their functions. We detected four pathways in various subsets of CD8^+^ T cells, including MIF, PARs, CCL, and VISFATIN (Fig. 3E), and four pathways in CD4^+^ T cell subsets, including MIF, VISFATIN, IL-16, and LIGHT (Fig. 3I). Thus, different cell subsets or the same cell subsets in different cells can rely on the same signaling network. For example, both CD4^+^ effector-GNLY and CD8^+^ effector-GNLY cells showed the pattern3 afferent pattern, which is linked to the VISFATIN signaling pathway.

### Identification of genes affecting T cell exhaustion.

**FIG 4:**
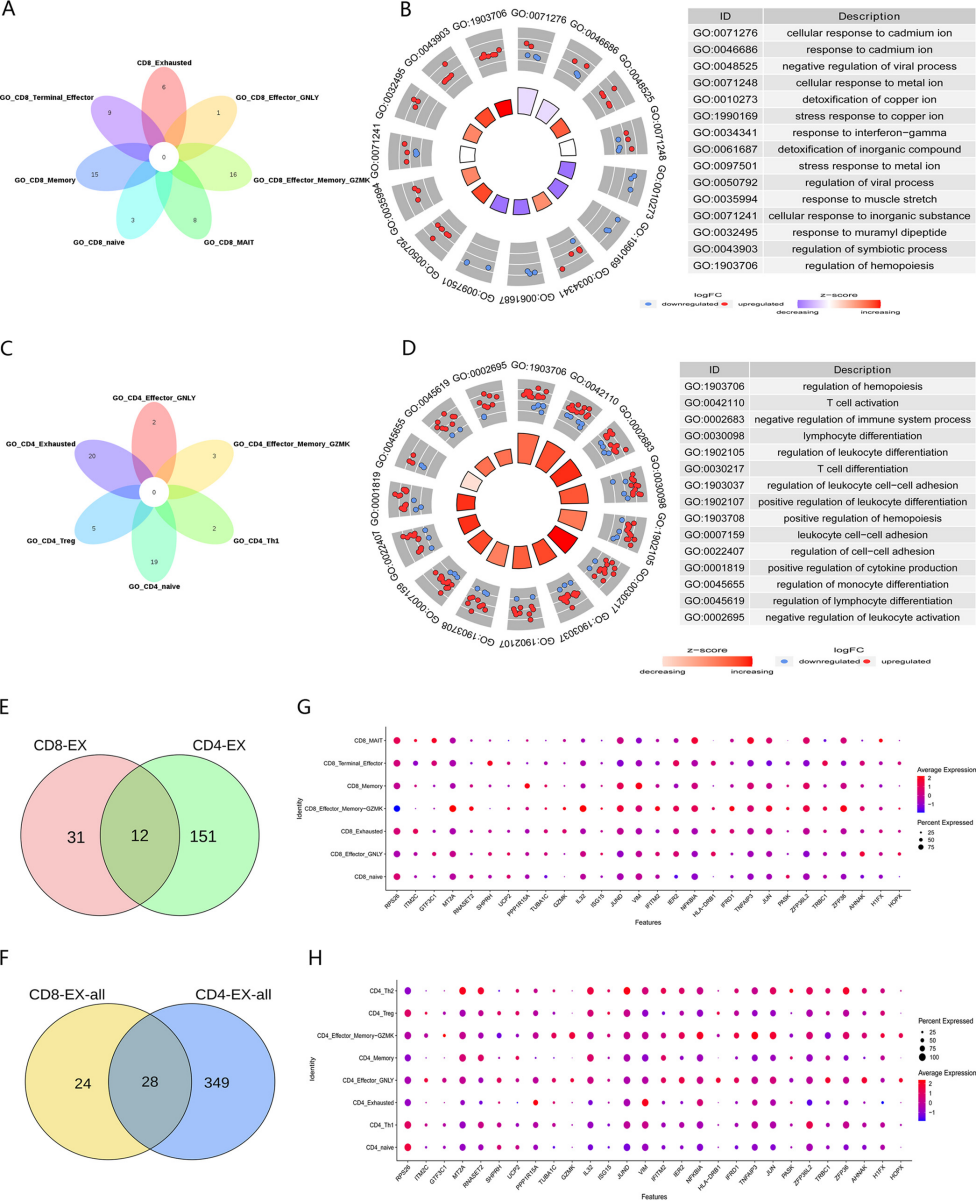
Common features of exhausted cells. (A) Venn diagram showing the intersection of biological processes of each CD8^+^ T-cell subset. (B) Chordal diagram showing the biological process enrichment of CD8^+^ T-cell exhaustion subsets. (C) Venn diagram showing the intersection of biological processes of each CD4^+^ T-cell subset. (D) Chordal diagram showing the biological process enrichment of CD4^+^ T-cell exhaustion subsets. (E) Venn diagram showing the intersection of genes with significant differences between CD8^+^ and CD4^+^ T-exhausted cells. (F) Venn diagram showing the intersection of differentially expressed genes of CD8^+^ and CD4^+^ T-exhausted cells. (G and H) Dot plots showing the cell expression ratios of T-cell exhaustion genes in each subset of CD8^+^ T cells (G) and CD4^+^ T cells (H).

We analyzed the function of each cell subset of CD8^+^ and CD4^+^ T cells. We crossed the top 20 pathways of CD8^+^ and CD4^+^ T cell subsets (Fig. 4A, C). In addition, we focused on the function of the exhausted subgroup. In CD8^+^ T-exhausted cells, the most significantly enriched pathway was the response to metal ions (Fig. 4B). However, these same pathways were also reflected in other subgroups. Because there were two cell subsets with fewer cells in the healthy group, only six CD4^+^ T-cell subsets were analyzed for enrichment. The main enrichment pathways of the CD4^+^ T-exhausted cell subsets were related to regulating the activation and differentiation of various immune cells (Fig. 4D). In summary, the functions of CD8^+^ and CD4^+^ T-exhausted cell subsets were notably different.

We compared the differential genes of CD8^+^ and CD4^+^ T-cell exhaustion subsets, and found 12 genes that may be related to T-cell exhaustion after TB infection (Fig. 4E, Table S3). We then expanded the screening scope and calculated the number of differentially expressed genes using the default parameters of the “FindMarker” function. CD8^+^ and CD4^+^ T-exhausted cell subsets had 28 identical genes (Fig. 4F, Table S3). By calculating the expression levels of these genes in each subgroup, we deduced that *ITM2C* might serve as an exhaustion marker gene in CD8^+^ T cells (Fig. 4G), whereas *H1FX*, *ZFP36L2*, *VIM*, and *PPP1R15A* might serve as exhaustion marker genes in CD4^+^ T cells (Fig. 4H).

### Identification of specific subsets of CD8^+^ T exhausted cells.

**FIG 5:**
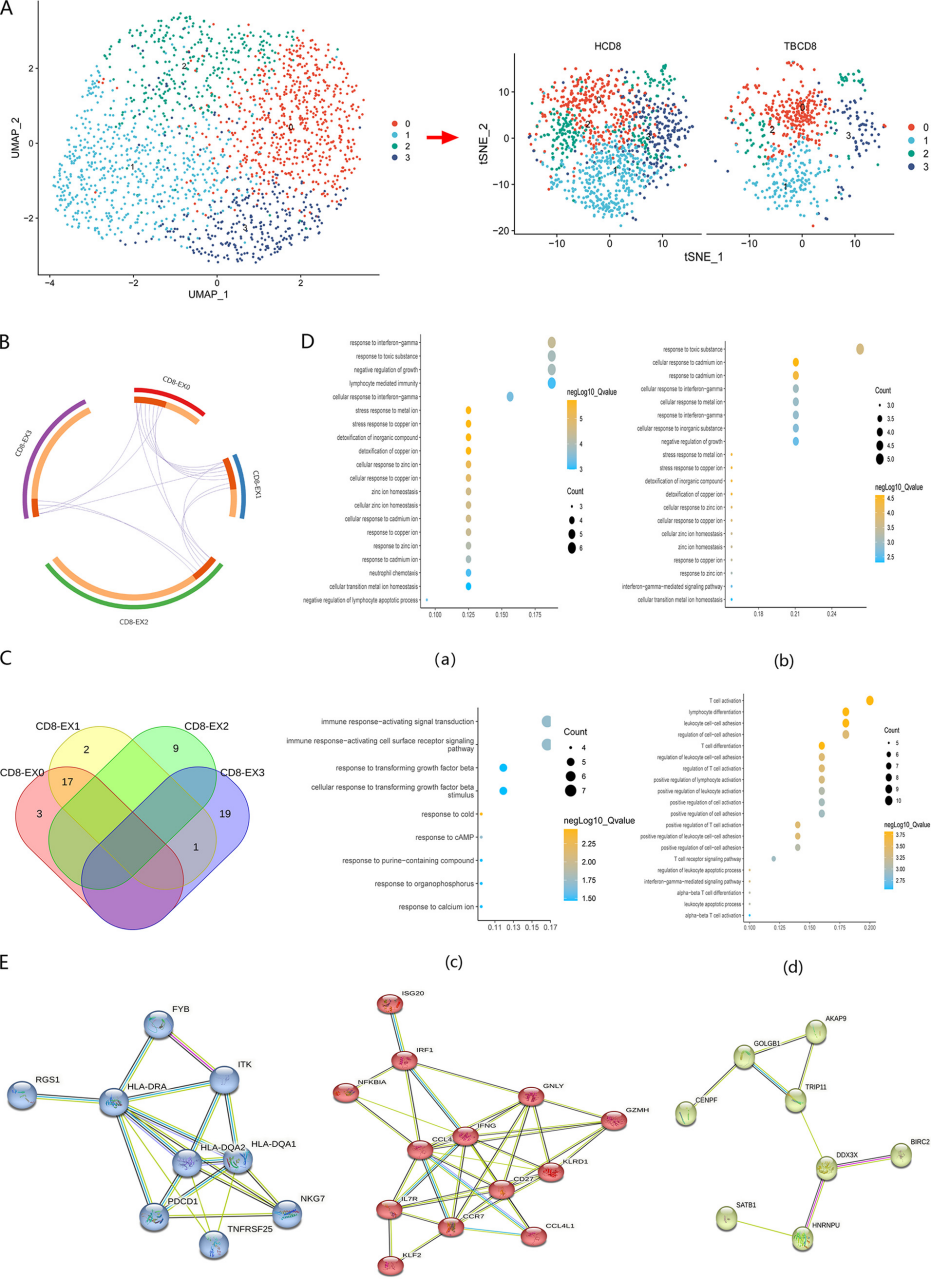
Dissection of CD8^+^-exhausted T cells. (A) UMAP projection diagram (left) showing the distribution of CD8^+^-exhausted T-cell subclusters. Each dot represents a single cell colored according to the cell clustering information. T-SNE diagrams (right) showing CD8^+^-exhausted T cell subclusters across the TB and healthy donor groups. (B) Circos diagrams showing the relationships of overlapping differentially expressed genes for each CD8^+^ T-exhausted cell subset. The purple trajectory lines link common genes shared by multiple clusters. Marker genes shared by multiple clusters are colored red, whereas orange labels are unique. (C) Venn diagram showing the intersection of biological processes of each cell subset. (D) Biological processes corresponding to each subcluster are as follows: (a) EX-0 cell subset; (b) EX-1 cell subset; (c) EX-2 cell subset; (d) EX-3 cell subset. (E) PPI network showing three key gene modules.

Furthermore, we observed distinct genetic signatures in exhausted cell subpopulations and identified four subclusters of CD8^+^ exhausted T cells (Fig. 5A). Some subclusters also changed in number before and after infection. Notably, in contrast to CD4^+^-exhausted T cells, we observed that the cell proportions of samples from patients with TB were significantly exhausted, and each subcluster exhibited relatively individual features. The marker genes were also different for each subcluster, with a high number of unique genes among the differential genes (Fig. 5B).

Furthermore, we analyzed the function of each cell subcluster (Fig. 5C and D). The main enrichment pathway of the CD8-EX0 and CD8-EX1 cell clusters was the response to metal ions, including copper, cadmium, and zinc ions. CD8-EX3 cell subsets enriched the activation and regulation of various immune cells. In a previous study, we found that CD8^+^ T-exhausted cells were responsible for cell activation and differentiation (16), which may be more related to this subgroup. In addition, compared with other pathways, CD8-EX3 was more strongly associated with immune-related pathways and genes, indicating a higher immune-related function. We speculated that CD8-EX3 could serve as a specific marker subset of CD8^+^ T-exhausted cells. In addition, three gene modules were extracted from CD8-EX3 using protein–protein interaction (PPI) networks based on the expression level (Fig. 5E). These modules may play an important role in cell exhaustion caused by TB infection.

## DISCUSSION

Our understanding of the human immune response mechanisms in TB infection remains limited because of a lack of information on the overall immune response. Identifying the changes in immune cells in response to infection can provide a better understanding of the effects of *Mtb* on the host immune system. With the development of sequencing technology, single-cell technology can achieve a more accurate grouping of cells by obtaining and analyzing the gene expression profile of each cell type (17, 18). This method has been applied to study the heterogeneity of pathogen infections and pathogen–host interactions (19 to 21). In this study, we characterized the transcriptome of CD4^+^ and CD8^+^ T-cell subsets isolated from PBMCs of patients with TB and healthy donors using single-cell sequencing. Because there are great differences in gene expression and immunity between males and females (22, 23), we only analyzed samples from individuals of the same sex. Furthermore, we analyzed each sample and found that the infection caused changes in cell subsets. Our study provides a panoramic analysis of the effects of *Mtb* infection on two distinct subsets of the most critical immune cells with a specific profile of exhausted T cells. These results will facilitate future studies in elucidating the impact of TB infection on the host.

The importance of T cells in controlling *Mtb* infection has always been under discussion (24). We analyzed the function of each subgroup using different methods to observe their heterogeneity. The biological pathway activity of many cell subsets had decreased. In the functional study of cell subsets, we found that the metabolic activities of many subsets changed after *Mtb* infection. The metabolic system plays an important role in maintaining the homeostasis of the host. Disturbances in host metabolism may favor the development of TB (25). Because of the biodynamic requirement for effective immune activation, *Mtb* can disrupt host immune defenses by interfering with the metabolic system (26). Therefore, targeting host or metabolic pathways may be an effective way to develop novel TB treatments and slow down the rate of cellular exhaustion. To that end, we plan on performing metabonomics analyses in our future work.

T-cell exhaustion can cause immune deficiency. CD4^+^ T-cell exhaustion can aggravate acute *Mtb* infection and affect CD8^+^ T function (27). CD4^+^ T cells help to prevent CD8^+^ T cells from being exhausted and have a synergistic effect on the control of *Mtb* infection. The depletion of CD8^+^ T was also observed in the lesions of Lepromatous leprosy (28). In a study of subgroup changes after HIV infection, we found through single-cell sequencing that the Treg subgroup also highly expressed some exhaustion marker genes (29). This is consistent with the phenomenon identified in the CD4^+^ T-cell subsets after TB infection in our study. Therefore, it is important to analyze the characteristics of exhausted cell subsets.

We analyzed the communication relationships between exhausted cell subsets and other subsets. The interactions between cells are very important for many biological processes (30). Cell-to-cell communication based on scRNA-seq can be used to reveal the deeper mechanism of diseases (31). The MIF signaling pathway is the most relevant pathway for importing depleted CD8^+^ T-cell subsets. It can regulate Toll-like receptor 4 to affect innate immunity and can also promote an inflammatory environment (32, 33). When connected with p53 and NF-κB signals, it can also affect cell senescence (34). These may all be factors that affect exhaustion.

The functional pathways with higher enrichment degrees of each cell subgroup had different purposes. The functions of the two exhausted subgroups were also quite different. But we found that the exhausted subpopulation was still in a state of cellular activation, consistent with the results of previous studies (16). In addition, we identified 12 genes that may affect cell exhaustion after TB infection in both CD4^+^ and CD8^+^ T cells. We also identified five genes that may be used as markers of cell exhaustion (one for CD8^+^ T cells and four for CD4^+^ T cells), with differential levels in the exhausted subpopulation from those in other subpopulations. These marker genes have not previously been related to cell exhaustion. For example, *H1FX* has mainly been highlighted to be associated with cancer to date (35, 36), whereas *ZFP36L2* was reported to induce apoptosis and inhibit cell proliferation (37, 38). VIM is involved in cell attachment, migration, and signal transduction (39). Here, we separated the exhausted subsets of CD4^+^ and CD8^+^ T cells into distinct subclusters via further unsupervised analysis. The exhausted T cells after *Mtb* infection may be composed of subclusters of cells in different states with distinct biological functions. There was no particularly prominent subcluster of CD4^+^ T cells (Fig. S5); however, we identified CD8-EX3 as a specific marker subcluster in CD8^+^ T cells. We also identified some gene modules that were critical for CD8^+^ T cell exhaustion, including the known gene *PDCD1* and our newly identified genes. Exploring the genes and pathways in the gene module can further narrow the screening of genes related to exhaustion. Therefore, our analyses identified some potential biomarkers associated with exhausted T cells. Further experiments should be conducted to validate these associations.

In the past, many studies on TB have also used single-cell sequencing technology, for example, by mapping the lung immune landscape of nonhuman primates to reveal the characteristics of different disease states and the correlation between the cells controlling the disease (40, 41). Many studies on TB primarily focus on macrophages, while paying less attention to the T cells in peripheral blood (42). The first scRNA-seq study on peripheral blood in TB showed that cytotoxic natural killer cell subsets were exhausted after infection and that this subset could be used as a marker to distinguish infection status (14). However, there has been no clear analysis of exhausted CD4^+^ and CD8^+^ T-cell subsets. Our study on the identification of depleted T cell subsets in healthy controls and patients with TB provides further evidence for the role of specific cell subsets in the progression of TB disease. In conclusion, our findings highlight the effects and mechanisms of T lymphocyte exhaustion on the host immune response to *Mtb*, which can inform the development of novel TB therapeutic methods.

## MATERIALS AND METHODS

### Ethics statement.

This study was approved by the Institutional Review Board of the Jilin University. All participants provided written informed consent. All experiments were performed in accordance with approved ethical and biosafety protocols.

### Collection of clinical samples.

Whole-blood samples were collected from patients with TB admitted to the First Hospital of Jilin University. Inclusion criteria for patients with active TB infection included HIV-antibody negative; exclusion of autoimmune diseases, diabetes, and other chronic infections; sputum acid-fast stained smear (+) and/or sputum mycobacterial culture (+); and a chest X-ray or computed tomography scan with features of active pulmonary TB. Most importantly, none of the patients had received anti-TB treatment prior to inclusion. The inclusion criteria for the healthy donors were no clinical evidence of TB infection, a negative T-SPOT.TB test, and no anti-TB treatment. All patients signed an informed consent form for participation.

### PBMC isolation.

PBMCs were obtained via gradient separation of whole-blood samples over a Ficoll-Hypaque density gradient (Ficoll-Paque Plus; Amersham Biosciences) with shaking at 2,000 rpm for 20 min at room temperature.

### Fluorescence-activated cell sorting.

Cells were labeled with fluorescein isothiocyanate-conjugated anti-human CD3 (Biolegend, 300305), phycoerythrin/Cyanine7-conjugated anti-human CD4 (Biolegend, 317413), and allophycocyanin-conjugated anti-human CD8 (Biolegend, 344721) antibodies for 30 min in a sorting buffer. The PBMCs were resuspended by adding 3 mL of phosphate-buffered saline (PBS) wash solution and centrifuged at 1,650 rpm for 5 min, and then a fluorescence-activated cell sorter was added and cell sorting was performed. The abundance of relevant cell populations was determined to be greater than 99%. Data analysis was performed using FlowJo software.

### ScRNA-Seq data processing and quality control.

Sorted cells were resuspended and washed with PBS, and the cell viabilities were detected and counted. The transcriptome and immune characteristics of CD4^+^ and CD8^+^ T cells were detected using a 10× genomics platform, according to the manufacturer’s instructions. Subsequently, CellRanger version 3.0.1 was used to map the raw reads to the GRCh38 human reference genome and extract the gene expression matrix, with default parameters. The CellRanger pipeline processing results were plugged into the Seurat version 4.0 workflow. Quality control was performed to filter out low-quality cells, with parameters, including mitochondrial gene-unique molecular identifier (UMI) proportions of <15% and gene mapped counts of >200.

### Identification of cluster markers and T-cell subsets.

Based on the Seurat version 4.0 workflow, we used the “NormalizeData” function to standardize the data and the “FindVariableFeatures” function to find hypervariable genes. Principal-component analysis (PCA), Uniform Manifold Approximation and Projection (UMAP), and t-distributed stochastic neighbor embedding (t-SNE) calculations were performed using the “RunPCA,” “RunUMAP,” and “RunTSNE” functions, respectively, which calculated the marker genes using “FindAllMarkers.” The mean expression level of the target gene set in the cell subsets was calculated using the “Mean Expression” function. Data fusion was performed using the “SelectIntegrationFeatures,” “FindIntegrationAnchors,” and “IntegrateData” functions.

### Analysis of cell communication.

We identified overexpressed ligands or receptors in cell groups using the “identifyOverExpressedGenes” function in CellChat 1.5.0. The overexpressed ligand–receptor interaction was identified by “identifyofexpression interactions.” We then used “projectData” to project the gene expression data. The communication probability was calculated, communication network was inferred via the “computeCommunProb” function, and the signal level was inferred by the “computecommuninproppath” function. The contribution of the ligand–receptor interaction to the overall signaling pathway was determined using the “netAnalysis_contribution” function. The signal roles and main contribution signals of cell groups were identified using “net analysis _ compute centricity.” The specified communication mode was then determined using the “identification communication patterns” command (43).

### Identification of differentially expressed genes and cell cycle distribution.

Differentially expressed genes were also identified using the “FindMarkers” function with the “Wilcox” method and by setting the logfc.threshold to log_2_(1.5). Genes with a fold change of ≥1.5 and adjusted *P* values of <0.05 were screened out. Cell cycle statements were classified using “CellCycleScoring.”

### Gene functional enrichment analysis.

Using GSEABase version 1.38.2 (44), ClusterProfiler version 3.18.1, and the (Metascape server [45]), we performed GSVA, and Gene Ontology (GO) and Kyoto Encyclopedia of Genes and Genomes (KEGG) annotation, with default parameters. Hallmark Gene Sets for GSVA were obtained from msigdbr 7.5.1. The PPI network was constructed using the STRING server version 11.5 (https://cn.string-db.org/).

### Statistical analysis.

An unpaired *t* test was used to analyze the differences between two groups. A *P* value of <0.05 was considered statistically significant.

### Data availability.

The data of this study are openly available in the Genome Sequence Archive of the Beijing Institute of Genomics (BIG) Data Center, Chinese Academy of Sciences, under reference number HRA002654.
