# Supplementary material for: Landscape of Exhausted T Cells in Tuberculosis Revealed by Single-Cell Sequencing
Source: Microbiol Spectr. 2023 Mar 14;11(2):e02839-22. doi: 10.1128/spectrum.02839-22 (PMC10100881; doi:10.1128/spectrum.02839-22)
Supplement: Supplemental file 4 — Fig. S1 to S5. Download spectrum.02839-22-s0004.pdf, PDF file, 10.7 MB [file spectrum.02839-22-s0004.pdf]

Supplementary Materials

Supplementary Figure S1. Data quality control. (A-B) Threshold screening of gene expression and mitochondrial gene content of $CD8^+$ T cells from healthy donors (A) and tuberculosis patients (B). (C) The fitting degree of correlation between $CD8^+$ T cells in two states was reflected by a PCA scatter plot. (D-E) Threshold screening of gene expression and mitochondrial gene content of $CD4^+$ T cells in healthy donors (D) and tuberculosis patients (E). (F) The fitting degree of correlation between $CD4^+$ T cells in two states was reflected by a PCA scatter plot.

Supplementary Figure S2. Single sample data processing. (A-B) The t-SNE projection of HC (A) and TB (B) of $CD8^+$ T cells. Each dot corresponds to a single cell, coloured according to cell subsets. (C-D) The heatmaps showing the top marker genes across each CD8+T-cell clusters of HC (C) and TB (D). (E-F) The t-SNE projection of HC (E) and TB (F) of $CD4^+$ T cell. Each dot corresponds to a single cell, coloured according to cell subsets. (G-H) The heatmaps showing the top marker genes across each $CD4^+$ T-cell clusters of HC (G) and TB (H).

Supplementary Figure S3. Single-cell transcriptomic clustering for $CD4^+$ and $CD8^+$ T cells. (A–B) The t-SNE plots showing the original $CD8^+$ (A) and $CD4^+$ (B) T-cell clusters. Each dot represents a single cell, and the clusters were labelled with individual colours. (C–D) The heatmaps showing the top 10 marker genes across each $CD8^+$ (C) and $CD4^+$ (D) T-cell clusters. (E-F) Dot plot of subset marker expression levels across each $CD8^+$ (E) and $CD4^+$ (F) T-cell subset. The dot sizes represent the expression percentage of selected cell markers in each T-cell subset, and the dot colours represent the average expression levels of selected markers.

Supplementary Figure S4. Cell cycle analysis. (A) UMAP projection of cell cycle of $CD8^+$ T cell subsets. (B) Stacked bar chart showing the proportion of each subgroup of $CD8^+$ T cells in each cell cycle. (C) UMAP projection of $CD8^+$ T cell subsets in each cell cycle. (D) UMAP projection of cell cycle of $CD4^+$ T cell subsets. (E) Stacked bar chart showing the proportion of each subgroup of $CD4^+$ T cells in each cell cycle. (F) UMAP projection of $CD4^+$ T cell subsets in each cell cycle.

Supplementary Figure S5. Dissection of $CD4^+$ exhausted T cells.UMAP projection diagram (left) showing the distribution of $CD4^+$ exhausted T-cell sub-clusters. Each dot represents a single cell coloured according to the cell clustering information. T-SNE diagrams (right) showing $CD4^+$ exhausted T-cell sub-clusters across the tuberculosis and healthy donor groups.

Supplementary Table S1. Summary of scRNA-Seq QC.

Supplementary Table S2. Characteristics of participating HCs and of individuals with TB infection.

Supplementary Table S3. Unique genes of depleted subgroups.

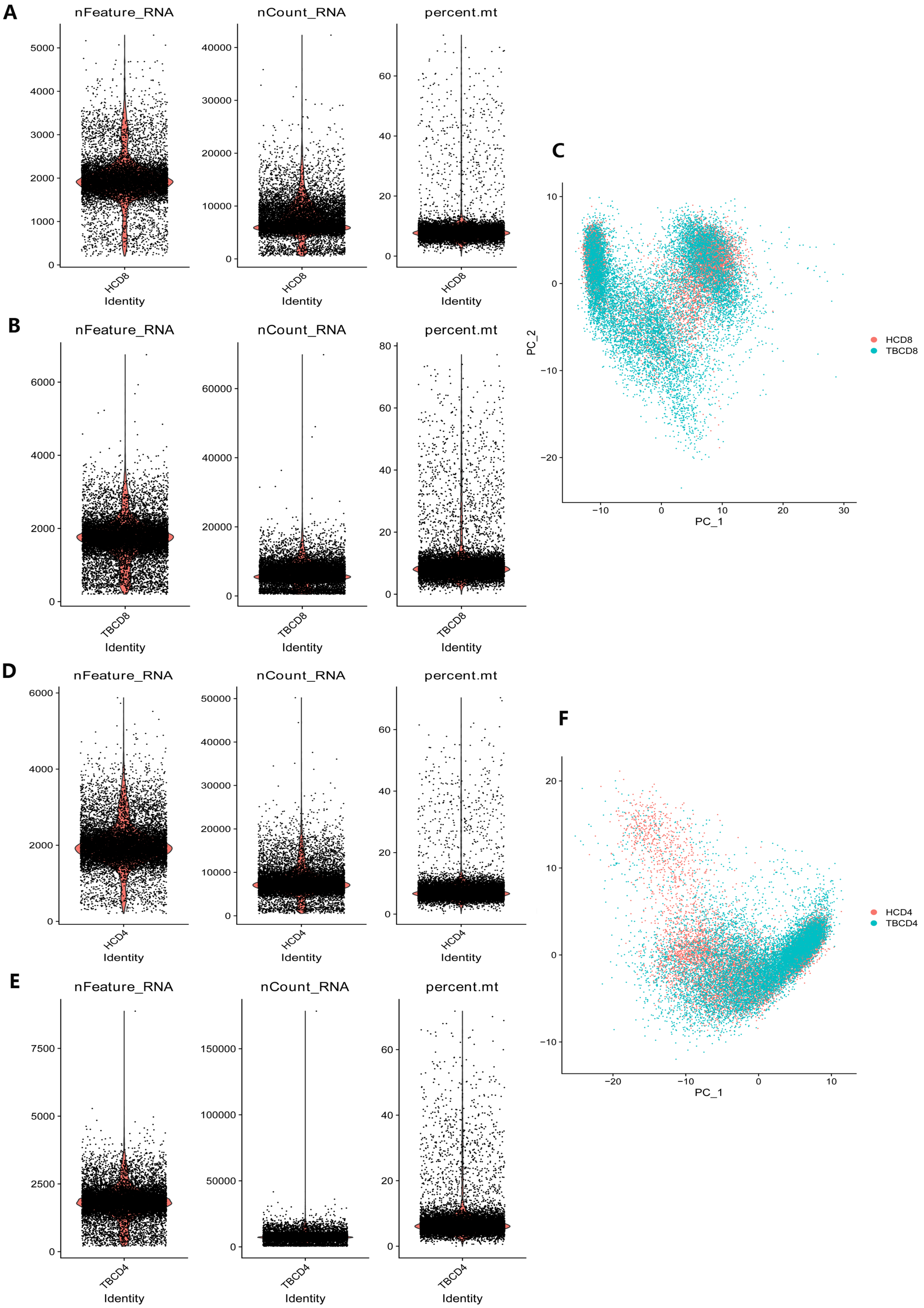

A
nFeature_RNA
nCount_RNA
percent.mt
HCD8
Identity
B
TBCD8
C
PC_2
PC_1
HCD8
TBCD8
D
HCD4
E
TBCD4
F
HCD4
TBCD4

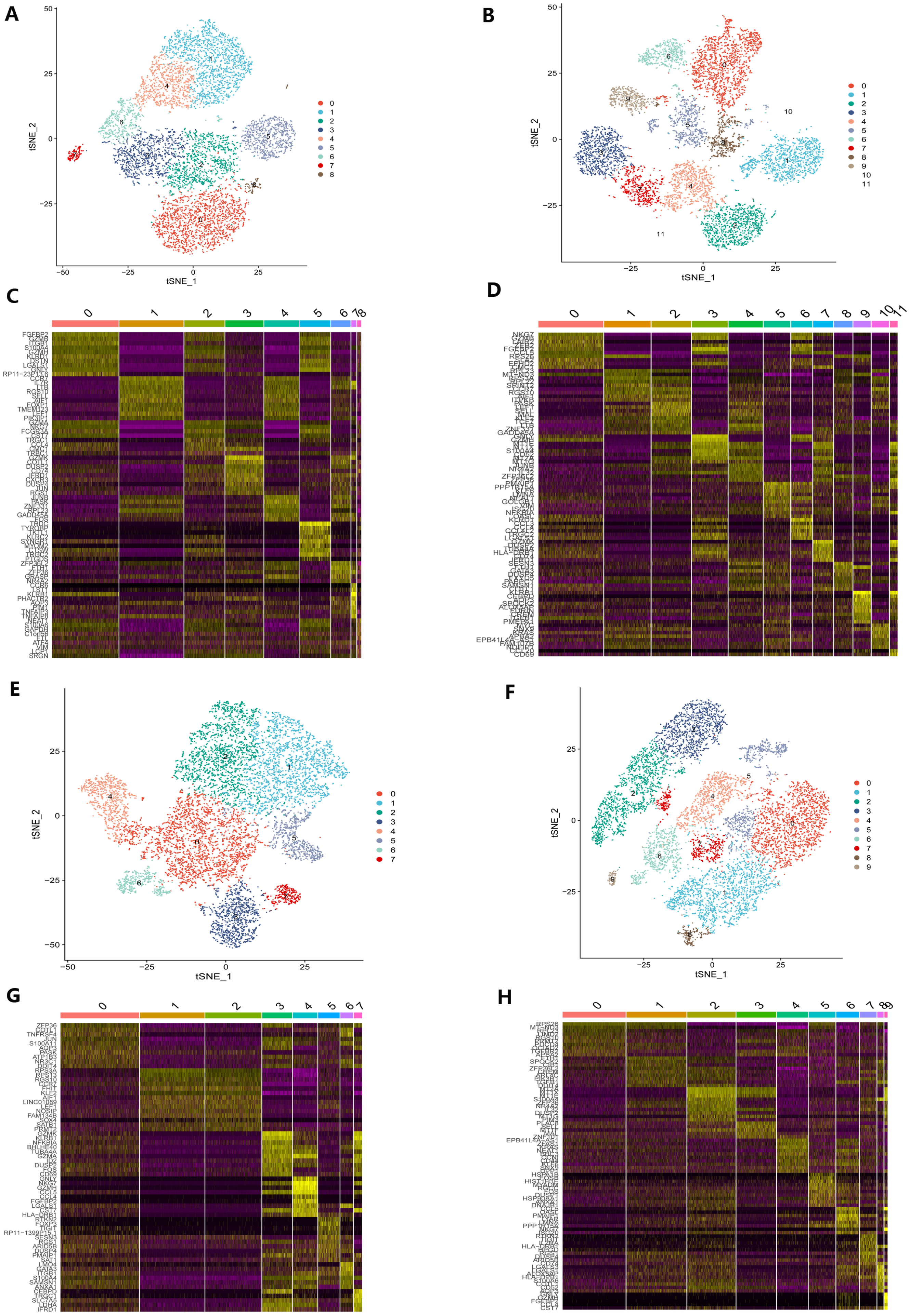
A
B
C
D
E
F
G
H
tSNE_1
tSNE_2

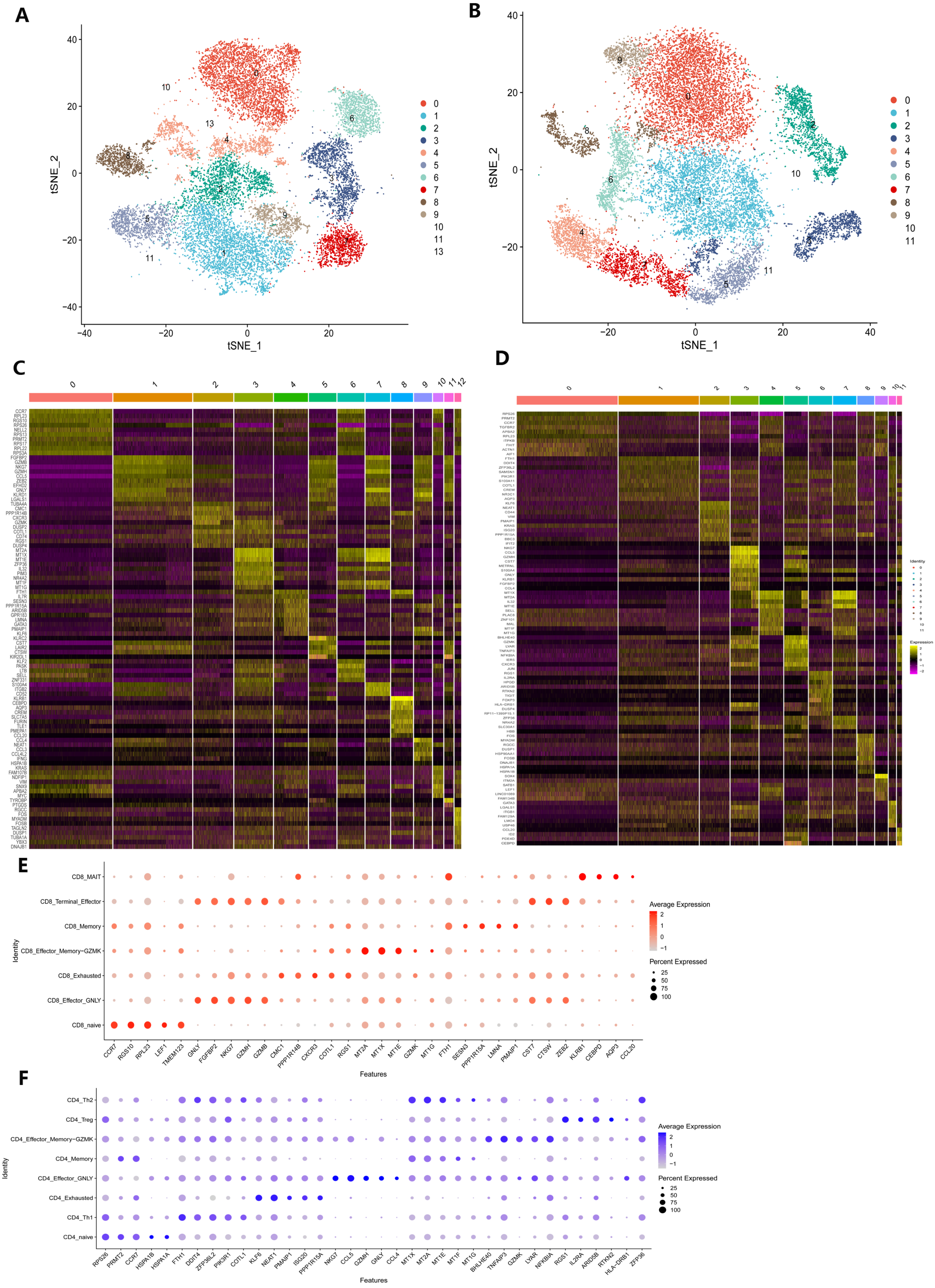
A
B
C
D
E
F
tSNE_1
tSNE_2
Identity
Expression
Average Expression
Percent Expressed
Features
CD8_MAIT
CD8_Terminal_Effector
CD8_Memory
CD8_Effector_Memory−GZMK
CD8_Exhausted
CD8_Effector_GNLY
CD8_naive
CCR7 RGS10 RPL23 LEF1 TMEM123 GNLY FGFBP2 NKG7 GZMH GZMB CMC1 PPP1R14B CXCR3 COTL1 RGS1 MT2A MT1X MT1E GZMK MT1G FTH1 SESN3 PPP1R15A LMNA PMAIP1 CST7 CTSW ZEB2 KLRB1 CEBPD AQP3 CCL20
CD4_Th2
CD4_Treg
CD4_Effector_Memory−GZMK
CD4_Memory
CD4_Effector_GNLY
CD4_Exhausted
CD4_Th1
CD4_naive
RPS26 PRMT2 CCR7 HSPA1B HSPA1A FTH1 DDIT4 ZFP36L2 PIK3R1 COTL1 KLF6 NEAT1 PMAIP1 ISG20 PPP1R15A NKG7 CCL5 GZMH GNLY CCL4 MT1X MT2A MT1E MT1F MT1G BHLHE40 TNFAIP3 GZMK LYAR NFKBIA RGS1 IL2RA ARID5B RTKN2 HLA-DRB1 ZFP36

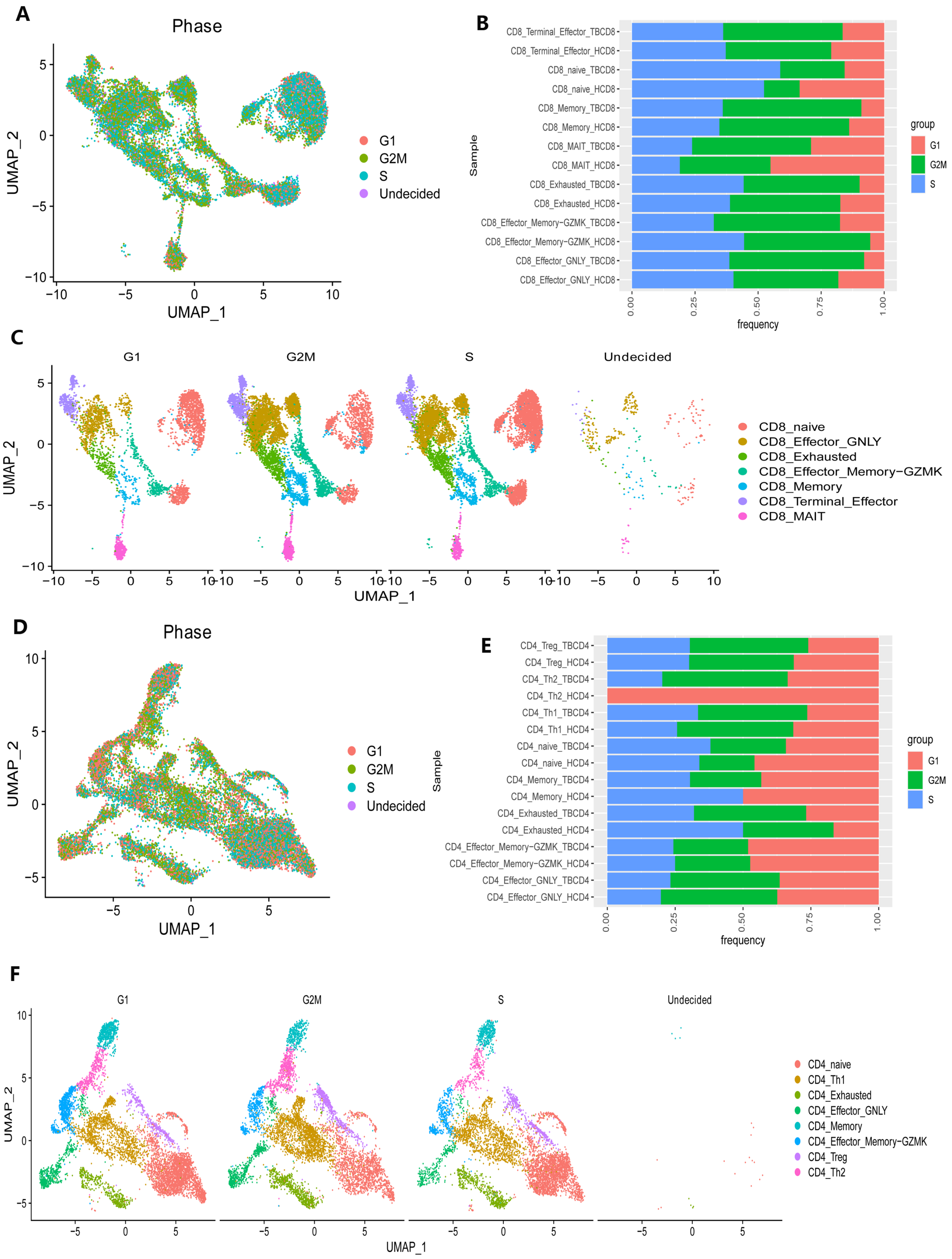
A
Phase
UMAP_2
UMAP_1
G1
G2M
S
Undecided
B
Sample
CD8_Terminal_Effector_TBCD8
CD8_Terminal_Effector_HCD8
CD8_naive_TBCD8
CD8_naive_HCD8
CD8_Memory_TBCD8
CD8_Memory_HCD8
CD8_MAIT_TBCD8
CD8_MAIT_HCD8
CD8_Exhausted_TBCD8
CD8_Exhausted_HCD8
CD8_Effector_Memory−GZMK_TBCD8
CD8_Effector_Memory−GZMK_HCD8
CD8_Effector_GNLY_TBCD8
CD8_Effector_GNLY_HCD8
frequency
group
G1
G2M
S
C
G1
G2M
S
Undecided
UMAP_2
UMAP_1
CD8_naive
CD8_Effector_GNLY
CD8_Exhausted
CD8_Effector_Memory−GZMK
CD8_Memory
CD8_Terminal_Effector
CD8_MAIT
D
Phase
UMAP_2
UMAP_1
G1
G2M
S
Undecided
E
Sample
CD4_Treg_TBCD4
CD4_Treg_HCD4
CD4_Th2_TBCD4
CD4_Th2_HCD4
CD4_Th1_TBCD4
CD4_Th1_HCD4
CD4_naive_TBCD4
CD4_naive_HCD4
CD4_Memory_TBCD4
CD4_Memory_HCD4
CD4_Exhausted_TBCD4
CD4_Exhausted_HCD4
CD4_Effector_Memory−GZMK_TBCD4
CD4_Effector_Memory−GZMK_HCD4
CD4_Effector_GNLY_TBCD4
CD4_Effector_GNLY_HCD4
frequency
group
G1
G2M
S
F
G1
G2M
S
Undecided
UMAP_2
UMAP_1
CD4_naive
CD4_Th1
CD4_Exhausted
CD4_Effector_GNLY
CD4_Memory
CD4_Effector_Memory−GZMK
CD4_Treg
CD4_Th2

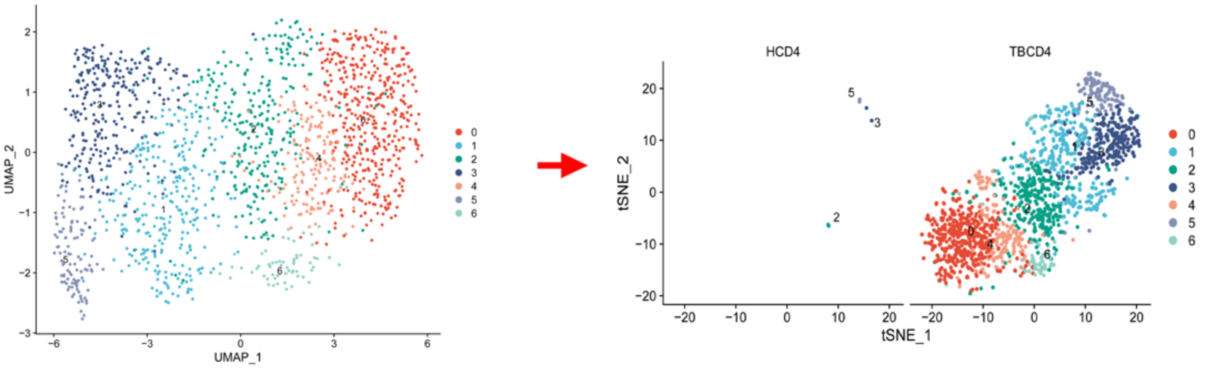
UMAP_1
UMAP_2
HCD4
TBCD4
tSNE_1
tSNE_2
0
1
2
3
4
5
6
